# A Systematic Review and Meta-analysis of the Use of High-Fidelity Simulation in Obstetric Ultrasound

**DOI:** 10.1097/SIH.0000000000000485

**Published:** 2020-09-14

**Authors:** Brian P. Dromey, Donald M. Peebles, Danail V. Stoyanov

**Affiliations:** From the Institute for Women's Health (B.P.D., D.M.P.), and Wellcome/EPSRC Centre for Interventional and Surgical Sciences (WEISS), University College London, London, United Kingdom (B.P.D., D.V.S.).

**Keywords:** Ultrasound, pregnancy, simulation, novice, expert, training, education

## Abstract

There is little global consensus on how to train, assess, and evaluate skills in obstetric ultrasound. The outcomes of curricula, where present, are often based on the number of clinical cases completed, rather than objective outcomes. The central question in this review is whether simulation enhances training and prepares trainees for clinical practice. A systematic review was conducted of the currently available literature in accordance with Preferred Reporting Items for Systematic Reviews and Meta-Analyses guidelines. Studies considering the use of simulators in training or assessment of sonographers were eligible for inclusion. We conclude that simulation is best used for acquisition of technical skills and image optimization. Best outcomes are observed when simulation augments traditional learning, with a strong focus on specific, objective, and measurable skills. Integrating simulation into training curricula could allow trainees to contribute to clinical service while learning. How skills learned in a simulated environment translate to the clinic is poorly addressed by the literature.

Ultrasound is a flexible, cost-effective investigation, which can be performed at the patient bedside. Despite these advantages, ultrasound is known to be operator dependent and have high interoperator variability.^1^ Training and competence assessment are of great importance to ensure safe clinical practice. In obstetrics, ultrasound can be used in acute clinical care to perform basic tasks such as confirmation of the fetal heartbeat or assessment of fetal presentation. Away from the delivery suite, intermediate level skills, such as monitoring fetal growth and well-being, have a higher training demand and require ongoing assessment of competency and quality assurance.^2^ Advanced applications include the diagnosis of major congenital abnormality, generally performed by doctors with a specialist interest in fetal medicine. A number of percutaneous, in utero, ultrasound-guided procedures are used to treat fetal anemia, congenital diaphragmatic hernia, and bladder outflow obstruction. A recent consensus statement considered US essential to the safe, timely, and effective practice of obstetrics and gynecology^3^ but acknowledged that training remains challenging. Given the wide variety of applications and that some techniques are performed at low frequency by highly specialized operators, a flexible, stepwise approach to skills training would seem the optimal solution. The consensus article concluded that *“Modern obstetrics and gynecology practice is virtually impossible without the use of ultrasound.”*^4^ The authors continued, *“it is clearly desirable for all obstetricians and gynecologists to have been trained robustly in basic sonographic skills so that their scanning in antenatal and gynecological clinics and on the labor ward is both safe and reproducible.*” Although widespread use of ultrasound is desirable, training in ultrasound is a challenge and there is little global consensus on how to train, assess, and evaluate skills in obstetric ultrasound. Competence is not necessarily directly related to clinical experience. Tolsgaard et al^5^ remarked that some experienced clinicians did not display expert-like behaviors despite daily use of obstetric ultrasound in their clinical practice. The authors hypothesized that poor basic training may be a root cause of this, suggesting that the operators did not have the correct foundation to benefit from later clinical training. The authors further hypothesized that the expected improvement in performance was not seen because sustained, deliberate practice rarely occurs in clinical practice. Attempts have been made by organizations such as The International Society of Ultrasound in Obstetrics and Gynecology and others to standardize requirements across Europe. The differences in delivery of clinical service may partly explain why there has been little global standardization of training and performance assessment to date. Practice differs widely; in Germany and Italy, all obstetric ultrasound is delivered by obstetricians or doctors training in obstetrics. In the United Kingdom and Denmark,^4^ more than 90% of routine obstetric ultrasounds are performed by sonographers or midwives. Most doctors performing obstetric ultrasound are subspecialist in fetal medicine who do not, generally, perform routine screening.

Traditional teaching of ultrasound, such as surgery, has taken the form of “see one, do one, teach one,”^6^ initially under the supervision of a more experienced operator. The outcomes of curricula, where present, are often based on the number of clinical cases completed, rather than objective outcomes of competence.^7^ Contemporary training curricula have evolved in response to patient safety concerns, increasing medical subspecialization and reduced training hours because of working time regulations. There have been concerns that “the specialist of tomorrow” will have significantly less experience in advanced procedures at the completion of their training than their trainers had at an equivalent career stage.^8^ These concerns are not limited to obstetrics and have been raised in many specialties. Ultrasound examinations, much like laparoscopic surgery, require the operator to interpret a dynamic image produced by the 3-dimensional position and motion of the ultrasound probe by means of a 2-dimensional visual display. It is accepted that laparoscopic skill and performance metrics improve with training and experience.^9^ Similarly, it might be expected that an ultrasonographers' performance would improve with training and practice. It is hypothesized that as novices gain experience and familiarity with a technique that their performance evolves,^10^ this is often referred to as a learning curve. The reasons for this are complex, related to familiarity with the task at hand, the surgical equipment, its limitations, and an appreciation of normal anatomy.

Simulation has been proposed as a strategy to shorten skill acquisition time and to allow clinicians learn in a safe, blame-free environment. Ultrasound seems an ideal candidate, but uptake has been disappointing. This might be because little attention has been focused on how to effectively integrate simulation into modern training curricula. A recent survey of UK trainees in obstetrics and gynecology reported that 79% considered simulation essential for training in ultrasound and that 90% would participate in a formal simulation-based training program. When provided, 76% of trainees found the simulator useful for improving clinical skills. Fifty-four percent never, or rarely, used the ultrasound simulation facilities available to them, citing a lack of formal guidance, unawareness of facilities, inconvenient access times, clinical workload, and time pressures as barriers to participation.^11^

The aims of this review are to investigate the use of high-fidelity simulation in obstetric ultrasound, to identify its usability for learners, and to establish if the skills obtained in a simulated environment can be translated to improved clinical performance.

The central question in this review is: *do training tools enhance training and prepare trainees for clinical practice?*

The secondary questions are if skills can be transferred to the clinical setting and if transferred skills are robust and sustained in the medium and long term?

## METHODS

### Protocol and Registration

A systematic review was conducted of the currently available literature. The review was completed in accordance with the Preferred Reporting Items for Systematic Reviews and Meta-Analyses standards for quality of reporting systematic reviews.^12^ The protocol was registered on the International Prospective Register of Systematic Reviews^13^ database in February 2019 as, “High-fidelity ultrasound simulation in obstetric ultrasound. Serious training tools or gaming toys? A review of the current literature,” reference number CRD42019122974. The registered protocol is available on the Prospero database at https://www.crd.york.ac.uk/prospero/.

### Eligibility Criteria

Studies considering the use of simulators in the training or assessment of ultrasound operators were eligible for inclusion. The PICO (Population, Interventions, Comparisons and Outcomes) model was considered when designing the search strategy.^14^ The Population was considered to be any trainee in ultrasound, and these may be doctors or allied health professionals. Interventions considered suitable were any use of a simulator, either before commencing clinical training or concurrent with clinical training. Suitable comparators included cohorts not trained on simulators, either in a parallel or crossover design. Outcomes showing a positive, negative, or no correlation on performance after the use of ultrasound simulators were considered suitable for inclusion.

### Information Sources

The search strategy developed was intended to provide results of relevance to training in obstetric ultrasound, which was agreed between the named authors. The search was completed on October 30, 2018. The search strategy used 4 database search tools: PubMed, EMBASE, Scopus, and Web of Science. Publications for inclusion were identified using the search terms “Simulat*” & “Training” & “Obstetric*,” either as keywords or contained within the article tittle. The “obstetric*” wildcard was used to capture variations including “obstetrician,” “obstetrics,” and “obstetric.” “Simulat*” wildcard was used to capture variations such as simulated, simulation, and simulator. The search terms were combined using the Boolean operator “OR.” The search was limited to articles in English and duplicates were removed by the author (B.P.D.) as part of the screening procedure to assess full-text articles for inclusion. No further articles were identified by examining the bibliography of the articles read in full.

### Search

**FIGURE 1:**
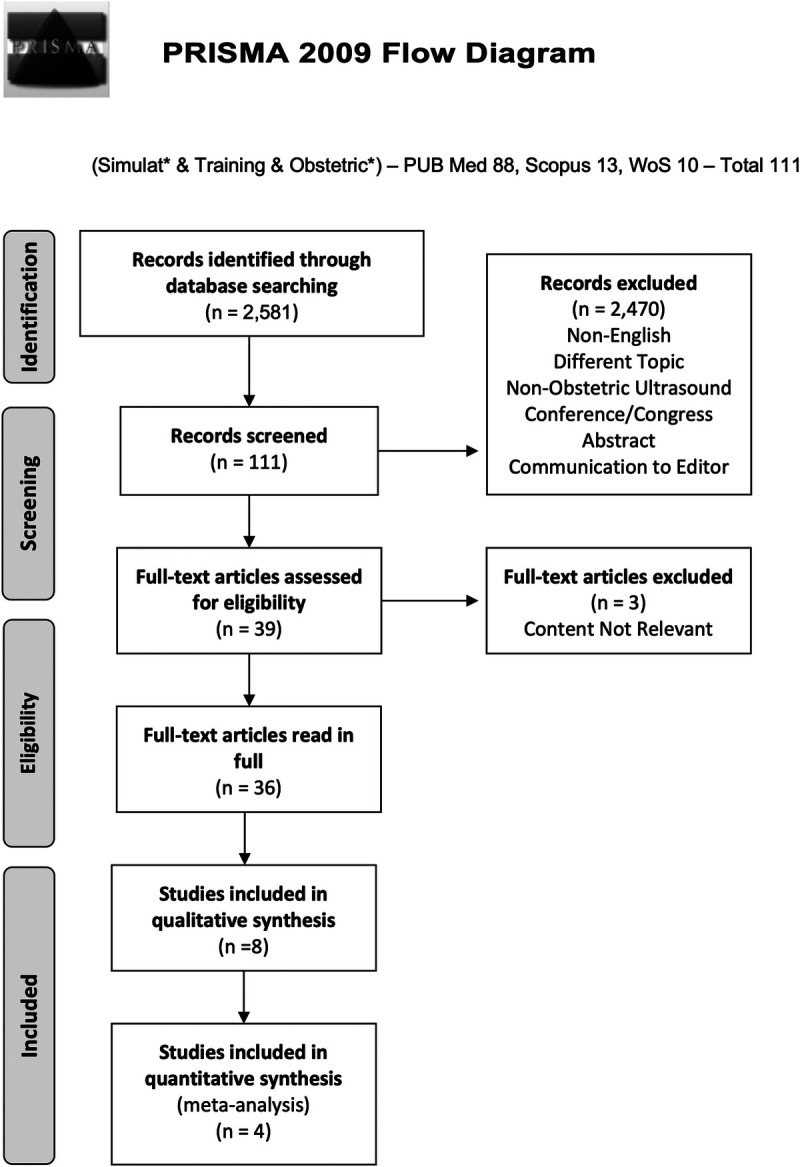
The search strategy undertaken. The Preferred Reporting Items for Systematic Reviews and Meta-Analyses flow chart is included as Figure 1.

The process is represented in Figure 1. A total of 2581 records were identified. A total of 2470 were excluded by screening the titles of the abstract. The reasons for exclusions were non-English, different topic, nonobstetric ultrasound, conference/congress abstract (full text not available), and communication to editor. From a pool of 2581 results, 111 were retrieved from the search engine results for screening. Once duplicates were excluded and abstracts were examined for relevance 39 articles were deemed suitable for inclusion. Three full-text articles were excluded as the content was not relevant to simulation in ultrasound.

### Study Selection

The remaining 36 articles were read in full. The motivation for this review was, as stated earlier, to determine whether the literature has reported behaviors, which could be used to establish the utility of simulators in obstetric ultrasound training. Studies that considered the use of high-fidelity simulators in ultrasound were considered for inclusion. The concept of “fidelity” refers to the realism of a particular simulator, how closely the simulator replicates the task being learned. All simulators replicate one, or more, parts of a clinical task for the purposes of education. High-fidelity simulators generally have some degree of computer control, interactivity, or trainee feedback. High-fidelity simulators are thought to increase realism and to have greater educational value because of this. Although there is wide variation in the design of ultrasound simulators, all are, by their nature, high-fidelity simulators. No studies were excluded based on the type of simulator used.

Studies examining the use of simulators in obstetric ultrasound or systematic reviews on the topic were eligible for inclusion. All of the included studies included novice operators. Study design was varied. Authors chose to compare novice and expert performance when using a simulator, whereas others chose to observe novice behavior before and after using a simulator. Studies were not excluded based on the type of medical professional selected to form the novice/inexperienced group as we recognize that obstetric ultrasound is performed by clinicians from a variety of backgrounds, including radiology, obstetrics, midwifery, and sonographers.

No studies were excluded based on their date of publication, as commercially available, high-fidelity ultrasound simulators are relatively new to the market. All studies were published between 2002 and 2018.

Studies were excluded if their primary outcomes were not in obstetric ultrasound. Studies were also excluded if the study did not include an educational intervention using a simulator. Although ultrasound validation studies were included in the qualitative analysis, these were excluded from the quantitative analysis as the primary outcome measured simulator performance rather than the learners' change of performance.

### Data Collection Process

Two researchers independently reviewed the 36 full-text articles. Discrepancies were resolved by discussion of the validity of the methods and quality of the continent within the article. After discussion, 8 studies^15–22^ were included in the qualitative analysis, 4 studies were included in the quantitative analysis and 4 studies did not report findings in a format suitable for inclusion in the meta-analysis.

### Data Items

A database of the 36 included articles was created using Microsoft Excel. For each full-text article read, the following data were recorded; title, author, article title, journal title, keywords, problem statement, research method, statistical methods used, number of included participants, author conclusions, findings in relation to past research, reviewer summary, and reviewer notes.

### Statistical Analysis—Risk of Bias

As part of the data collection and meta-analysis analysis process included studies were scored using the Medical Education Research Study Quality Instrument (MERSQI) tool.^23^ The MERSQI is an instrument developed for measuring the quality of education research studies. The maximum score is 18, made up from the flowing domains: study design (3), number of institutions sampled (1.5), follow-up (1.5), outcome assessment (3), validity evidence (3), data analysis (3), and outcome type (3). A score of 12 or higher is considered an indication of high study quality. The MERSQI authors describe their assessment of 210 medical education research studies published in 13 peer-reviewed journals. Over a 15-month period, the mean MERSQI score was 9.95 (SD = 2.34; range = 5–16). We calculated the mean MERSQI score for included articles of 11.88 (SD = 1.81; range = 9.5–15). In this context, the articles included are, at least, reflective of study quality seen in broader medical education.

### Statistical Analysis—Summary Measures and Synthesis of Results

**FIGURE 2:**
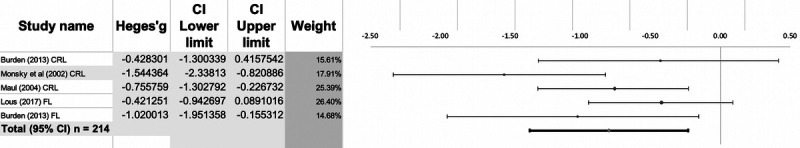
Forest plot diagram of meta-analysis. Four studies reported outcomes of fetal biometry, which were suitable for inclusion in the analysis.

Review Manager 5.3^24^ (The Cochrane Collaboration, 2014.) was used to produce forest plots of the included studies. Meta-essentials^25^ running on Excel (Microsoft Excel for Mac Version 16.32) was used to perform the meta-analysis and to calculate the sensitivity and specificity of each included study. The results are shown in Figure 2, finding favorable effect for improved accuracy of biometry in obstetric ultrasound after simulation training.

All the included studies had similar methodology and all included novice participants. In all studies, a group of novice operators was asked to complete a specified training package. Their performance was compared before and after completion of the training package. No study compared novice with expert performance, either before or after the training. No study compared objective clinical performance before and after training. All studies were competed in a training center, or simulation suite, and none were undertaken in a clinical area. Measures of heterogeneity indicated moderate heterogeneity. Cochrane *Q* value was calculated at 6.73.

Eight studies were included in the qualitative analysis, all 8 studies recruited doctors. None of the included studies recruited nurses, sonographers, midwives, or students. Five studies recruited doctors from obstetrics and gynecology,^15–17,20,22^ and the remaining studies recruited trainees in emergency medicine^16^ and radiology.^21^ One study recruited any postgraduate year 0–5 doctor.^18^ The calculated *I*^2^ value of 40% indicates moderate heterogeneity between the studies, despite difference in design, methodology, and reporting. In total, 6 models of simulator were used, UltraSim, VimedixTM US simulator, Canadian Amnio Model, Scantrainer, UltraSim, and SonoTrainer. A summary of the findings of the qualitative analysis is presented in Table 1.

**TABLE 1:** Summary of the Qualitative Analysis of the Included Manuscripts

Study Authors	Burden et al^15^	Todsen et al^16^	Chalouhi et al^17^	Pittini et al^18^	Jensen et al^19^	Madsen et al^20^	Monsky et al^21^	Maul et al^22^
Year	2013	2017	2016	2002	2018	2014	2002	2004
No. participants	26	30	29	30	25	28	16	45
No. experts	8			15	0	12	0	
No. novice	18	30	29	12	25	16	16	21
Purpose of the study	To assess the usability of VR simulation for obstetric US trainees.	assess progress made in the US measurement of FL by students after 1 H of training on US obstetric simulators.	To test the validity of an obstetrical US simulator as a tool for evaluating trainees following structured training	How to teach procedural skills without compromising patients health	To investigate the learning curves for novices training the FAST protocol on a VR simulator.	To assess the validity and reliability of performance measures	The purpose of our study was to evaluate the effectiveness of a sonographic simulator in evaluating residents before their taking overnight call.	To evaluate the effectiveness of the SonoTrainer ultrasound simulator as a training method for first-trimester screening
Participant group	Obstetrics and gynecology trainees	Obstetric US trainees	Obstetric US trainees	Doctors—PGY 0–5	Emergency medicine trainees	Obstetrics and gynecology trainees	Radiology trainees	Certified obstetricians
Study design and control	Comparative study, no control	Prospective single-center study	Comparative study, no control	Prospective single-center study	Case control	Comparative study, no control.	Case-control observational	Case-control with intervention applied. One control group
MERSIQ Score	11.5	10.5	11	11	13.5	15	9.5	13
Type of simulated training program (intervention)	Fetal biometry Early pregnancy	FL at 20 wk	LF, HC, AC. 4 Chamber, cardiac apex, heart crux, pulmonary vein, descending aorta, ROI.	Amniocentesis	TA USS (FAST)	TV USS	TV and TA USS	TA USS
Type of simulator	UltraSim	VimedixTM US simulator	Vimedix	Canadian Amnio Model	Scantrainer	Scantrainer	UltraSim	SonoTrainer
Trimester of pregnancy	1, 3	2	2	2	1	1	1	1
Time interval between training and testing	5 attempts. First and last measurements taken	Immediately before and after 1-H training session.	Nil. Training completed before attending for examination.	Immediately before and after training session.	Immediately before and after training session.	2 mo	10 H of self-directed study	Unclear -? Education modules completed.
Alternate training program as comparator to simulated training	Nil	Nil	Nil	Nil	Nil	Nil	Conventional clinical training	Theoretical training package
Outcome measures	Difference in time and accuracy of CRL, BPD, OFD, FL, and time between first and last scans	Time to obtain FL before and after training. FL measurement obtained.	Score-based evaluation of 6 morphological planes in fetal cardiac imaging	Score-based evaluation of US-guided amniocentesis	Time to achieve mastery learning level, corresponding to the performance level of a group of US experts.	Which metrics distinguish reliably between expert and novice	Mean test scores comparing 2 consecutive classes of 8 residents	Differences in NT, CRL, and mean duration per examination.

The table includes the stated purpose, design, and findings of each study.

BPD, Biarietal Diameter; NT, nuchal translucency; OFD,Occipito-Frontal Diameter; US, ultrasound; VR, virtual reality.

## RESULTS

The results of the meta-analysis find that superior performance has been achieved after training using high-fidelity ultrasound simulation. All the evaluated results considered performance before and after a training event using an ultrasound simulator.

As detailed in the methodology, 8 studies^15–22^ were included in the qualitative analysis. Five outcome measures from 4 studies were included in the quantitative analysis.^15,16,21,22^ In total, 214 participants were recruited to the 4 studies and 129 were novice participants (56%). All 4 studies reported positive effect on operator performance. Specifically, the performance improvements were noted in the measurement of crown rump length (CRL, reported in 3 studies) and in femur length (FL, reported in 2 studies). These improvements were seen, regardless of the model of simulator used. Across the 8 studies, 6 models of simulator were used.

All studies had similar aims, but the subsequent training or instruction differed. All studies established baseline performance for each user, and all studies did this using a simulator. All studies used a single model of simulator. The participants undertook assessment and training on the same model of simulator. Studies by Burden et al,^15^ Lous et al,^26^ Todsen et al,^16^ Chalouhi et al,^17^ Pitttini et al,^18^ and Jensen et al^19^ required participants to attend a single simulator session, and these studies did not compare simulator-based training to other training methods.

Madsen et al^20^ repeatedly assessed participants over 2 months, whereas Monsky et al^21^ required participants to compete 10 hours of self-directed learning using the simulator and compared final performance to doctors of similar grade who had not participated.

Three studies examined operator performance in the first trimester of pregnancy measuring the CRL. The remaining 2 studies examined performance in fetal biometry in the second trimester. One study specifically reported FL, but other measures of fetal biometry were not reported. Some studies used expert operators as a control group. One study compared the use of a high-fidelity ultrasound simulator to a theoretical training package, one study compared 10 hours of self-direct learning using the UltraSim to conventional clinical training.

As stated earlier, the aims of this review were to investigate the use of high-fidelity simulation in obstetric ultrasound, to identify its usability for learners, and to establish whether the skills obtained in a simulated environment can be translated to improved clinical performance, which is sustained over time. The articles included in the qualitative review have been scored against these aims in Table 2. The study design used by authors predominantly focused on the functionality and usability of ultrasound simulators. Most studies have not focused on how skills are translated from the simulation suite into the clinical environment, how the acquired skills translate to clinical practice, and if the skills are maintained over time.

**TABLE 2:** Tabulation of the Qualitative Analysis of Each of the Included Articles Against the Aims of the Review

Study Authors	Burden et al^15^	Todsen et al^16^	Chalouhi et al^17^	Pittini et al^18^	Jensen et al^19^	Madsen et al^20^	Monsky et al^21^	Maul et al^22^
Year	2013	2017	2016	2002	2018	2014	2002	2004
1. The use of high-fidelity simulation in obstetric ultrasound,	Article specifically used obstetric ultrasound.	Fetal biometry using obstetric ultrasound.	measurements of biparietal diameter, abdominal circumference, and FL as well as reference planes for cardiac 4-chamber and outflow tracts, kidneys, stomach/diaphragm, spine, and face.	Amniocentesis, not specifically examining ultrasound skills.	Simulator used, but for the FAST protocol. FAST protocol can be used to identify free fluid in the abdomen associated with Ectopic pregnancy.	Transvaginal US—mainly used in early pregnancy or gynecology.	General radiology.	Article specifically used obstetric ultrasound.
2. To identify usability for learners	The user questionnaire identified timetabling issues as a barrier to training.	All participants were preparing for national examination.	All participants were preparing for national examination.	A curriculum was constructed to allow trainees gain skill.	90% of participants completed the learning program.	Mapped performance metrics shipped with the system to expert performance.	Completion was mandatory at departmental level.	Comparison of a didactic training curriculum with an integrated alternative.
3. To establish if the skills obtained in a simulated environment can be translated to improved clinical performance.	Improved performance on the simulator, but no clinical correlation undertaken.	Shorter image acquisition time, higher skill and all trainees used zoom function after training in assessment.	Similar dexterity scores were achieved, higher scores for image quality in assessment.	Checklist and training improved performance in simulated setting.	The “mastery” level was benchmarked against experts asked to perform the FAST protocol on that particular simulator.	With time and repeated practice improved performance on the simulator was shown.	The participants had to be assessed as competent before being allowed to perform independent ultrasound in clinic.	The expert level was benchmarked against experts asked to perform ultrasound on that particular simulator.
4. Can skills be transferred to the clinical setting?	No clinical correlation undertaken.	No clinical correlation undertaken. Technique and image optimization better after training.	Not directly asked or answered.	Unclear.	Mastery level achieved by the trainees is based on expert performance.	Unclear.	Better performance was seen in the group exposed to simulator training than the group not given US simulator training.	Unclear—assumed.
5. If transferred skills are robust and sustained in the medium and long term?	No follow-up cohort.	No follow-up cohort.	No follow-up.	Assumed, but no follow-up of the trainees.	No follow-up.	No follow-up.	No statistical significance between 2 groups, but subjective reported assessment and trainee confidence improved.	No follow-up.

The use of simulators by learners and the motivations for learners to use the simulators have been considered by all authors. Some consideration has also been given to how the learner can be assessed in the simulated environment. Only Monsky et al^21^ considered how the skills acquired in the simulated setting compared with those acquired by learners who had not been exposed to simulation.

## DISCUSSION

All the included studies look to validate the concept of using simulation for training or assessment in obstetric ultrasound. This finding is reassuring and supports the uptake of simulation as a training methodology across many medical specialties. Our meta-analysis shows that skills can be acquired, improved, and assessed by means of a high-fidelity simulator. In particular, our findings suggest that simulation can be best used for acquisition of technical skills^15^ and image optimization.^27^ Superior technical ability may accelerate a learner's time to competence.^20^ Our review of the literature finds that simulation training can be used to equip novice ultrasound practitioners with sufficient skills to perform basic obstetric ultrasound in a clinical environment under direct supervision.

Our findings suggest that consideration ought to be given to integrate simulation training into the clinical curriculum. Even in research settings trainees reported clinical commitments as barriers to engaging with simulation training.^11^ The highest levels of engagement, 90%, were seen when participation was mandated by the faculty by Monsky et al.^21^ The authors undertook simulator-based assessment of radiology residents before taking overnight call. The authors were surprised to find that their findings challenged established beliefs within the radiology department that residents were suitably and adequately trained before taking up semiautonomous clinical practice. The participant survey also highlighted residents' concerns about their own preparedness for overnight calls. As a result, the authors modified the residency training program at their hospital. The redesigned curriculum addressed these concerns, and an additional 8 weeks of targeted, clinical training, focusing specifically on transvaginal ultrasound was provided. Twelve months later, the experiment was repeated. The authors found that residents performed significantly better on the simulator and reported higher confidence in performing ultrasound. Senior clinicians also reported higher subjective performance scores for residents when being assessed.

Studies by Chalouhi et al^27^ and Maul et al^22^ showed that even novice operators could achieve competent performance in obstetric ultrasound when being trained by means of simulation alone. The authors compared their simulation-based curriculum with conventional didactic teaching of ultrasound theory and practice.

The example of simulator use in pilot training is often used as justification for the use of simulation in medical education. It is true that high-fidelity simulators are universally used for training airline pilots. When considering the use of simulation in medicine, it is important to understand that full-motion flight simulators are integrated into pilot training, assessment, and licensing. Initial pilot training and recurrent assessment in a simulator take place every 6 months for commercial pilots. Mandatory emergency simulator sessions allow trainers to create an entirely immersive experience, recreating the systems and motion of the aircraft and the human factors, which have been recurrent contributors to accidents and near misses. None of the simulators described to date have addressed the clinical context in which the trainee will eventually work. The current devices focus on technical skills proficiency, while ignoring communication with patients and colleagues, distractions, and clinical management, which contribute to overall clinical performance. Our review finds that that trainees in obstetric ultrasound can benefit from the use of a high-fidelity simulator but that these tools are not formally integrated into medical education curricula. It is preferable that training programs be based on objective outcomes, rather than trainer reports and arbitrary numbers of cases recorded in a log book.

We suggest that high-fidelity ultrasound simulation can be used to train users more quickly; however, our study is limited by the heterogeneity of the evidence base. The wide disparity in maternal-fetal medicine (MFM) training curricula globally is reflected in the heterogeneity of the studies and reported outcomes. These limit the generalizability of our results, as we were able to include 4 studies and a total of 214 participants in the meta-analysis. Even with these limited numbers, we were able to show a positive effect for simulation training. The positive result may reflect that by using a simulator, the participants were gaining tuition and experience that they would not otherwise have been exposed to. The effects seen might be attributable to additional intentional practice, rather than the simulator itself. Because all studies carried out baseline assessment, training, and subsequent assessment on the same model of simulator, it is possible that the results reflect user familiarity with the simulator, rather than a true improvement in clinical skill. The limitations of the study highlight the need for future research to consider how skills acquired in the simulation setting translate to a clinical setting. Research methodology and study design need careful consideration, as pretest/posttest designs may overestimate the effect of the intervention.

Based on this literature review, our group is developing a longitudinal study to assess trainees using baseline scans on pregnant volunteers and then allow them to undertake a training package or clinical attachment. At the end of the attachment, the participants will be asked to undertake fetal biometry in a clinical setting. This will allow us to understand how skills obtained in a simulated environment can be translated to clinical reality and how robust skills are when presented with the variability inherent in obstetric scanning because of maternal habitus, stage of pregnancy, fetal presentation, and position.

## CONCLUSIONS

This review finds evidence of benefit for high-fidelity ultrasound simulation. The evidence for deployment in training is limited, but the authors have found their own training curricula challenged by the introduction of simulation-based training and assessment. In these instances, simulation has been used to augment traditional learning, with a strong focus on specific, objective, and measurable clinical outcomes, audit, and revision of the curriculum based on learner feedback.

Further investigation of ultrasound simulation in training should follow models closer to pilot training, were training, and ongoing assessment are routine, mandatory, and completed by all grades. The inertia to adopt simulation as a valid means of learning can be challenged by considering the design of further studies now that the utility and validation of this equipment are established.

Simulation is best considered as a waypoint to allow the learner to transition to semiautonomous practice in a supervised, clinical setting. By integrating ultrasound simulation into training curricula and promoting self-directed learning, trainees could contribute to the clinical service while learning a complex skill. Integrating ultrasound training into clinical workflow would allow us to establish if skills acquired in the simulated environment correlate with clinical performance and if skills are maintained in the longer term, which has been poorly considered by the literature to date.
